# Antitumor Profile of Combined *Matricaria recutita* Flower Extract and 5-Fluorouracil Chemotherapy in Sarcoma 180 In Vivo Model

**DOI:** 10.3390/toxics11040375

**Published:** 2023-04-14

**Authors:** Sara A. Santos, Ricardo G. Amaral, Ariel S. Graça, Silvana V. F. Gomes, Fabrício P. Santana, Iza B. de Oliveira, Luciana N. Andrade, Patrícia Severino, Ricardo L. C. de Albuquerque-Júnior, Sandra L. Santos, Eliana B. Souto, Adriana A. Carvalho

**Affiliations:** 1 Department of Physiology, Federal University of Sergipe, São Cristóvão CEP 49100-000, Brazil; 2 Institute de Technology and Research (ITP), Tiradentes University, Aracaju CEP 49100-000, Brazil; 3 Department of Medicine, Campus of Lagarto, Federal University of Sergipe, Lagarto CEP 49400-000, Brazil; 4 Department of Pathology, Federal University of Santa Catarina, Florianópolis CEP 88040-370, Brazil; 5 UCIBIO—Applied Molecular Biosciences Unit, MEDTECH, Laboratory of Pharmaceutical Technology, Department of Drug Sciences, Faculty of Pharmacy, University of Porto, 4050-313 Porto, Portugal; 6 Associate Laboratory i4HB—Institute for Health and Bioeconomy, Faculty of Pharmacy, University of Porto, 4050-313 Porto, Portugal; 7 Department of Pharmacy, Campus of Lagarto, Federal University of Sergipe, Lagarto CEP 49400-000, Brazil

**Keywords:** *Matricaria recutita*, 5-fluorouracil, antitumor, sarcoma 180 model, in vivo, chemotherapy

## Abstract

Medicinal plants have been commonly associated with chemotherapeutic treatments, as an approach to reduce the toxicological risks of classical anticancer drugs. The objective of this study was to evaluate the effects of combining the antineoplastic drug 5-fluorouracil (5-FU) with *Matricaria recutita* flowers extract (MRFE) to treat mice transplanted with sarcoma 180. Tumor inhibition, body and visceral mass variation, biochemical, hematological, and histopathological parameters were evaluated. The isolated 5-FU, 5-FU+MRFE 100 mg/kg/day, and 5-FU+MRFE 200 mg/kg/day reduced tumor growth; however, 5-FU+MRFE 200 mg/kg/day showed a more significant tumor reduction when compared to 5-FU alone. These results corroborated with the analysis of the tumor histopathological and immunodetection of the Ki67 antigen. In the toxicological analysis of the association 5-FU+MRFE 200 mg/kg/day, an intense loss of body mass was observed, possibly as a result of diarrhea. In addition, spleen atrophy, with a reduction in white pulp, leukopenia and thrombocytopenia, was observed in the 5-FU groups alone and associated with MRFE 200 mg/kg/day; however, there was no statistical difference between these groups. Therefore, the MRFE 200 mg/kg/day did not interfere in myelosuppressive action of 5-FU. In hematological analysis, body and visceral mass variation and biochemical parameters related to renal (urea and creatinine) and cardiac (CK-MB) function, no alteration was observed. In biochemical parameters related to liver function enzymes, there was a reduction in aspartate transaminase (AST) values in the 5-FU groups alone and associated with MRFE 200 mg/kg/day; however, there was no statistical difference between these groups. Therefore, the MRFE 200 mg/kg/day does not appear to influence enzyme reduction. The results of this study suggest that the association between the 5-FU+MRFE 200 can positively interfere with the antitumor activity, promoting the antineoplastic-induced reduction in body mass, while minimizing the toxicity of chemotherapy.

## 1. Introduction

The most common forms of cancer treatment are chemotherapy, radiotherapy, and surgery, which can be combined depending on the type of tumor to treat. Chemotherapy is especially used to kill neoplastic cells; however, it is strongly linked to high toxicity and severe side effects given the systemic distribution of the anticancer drugs [1,2]. The toxic effects reflect the inability of chemotherapeutic agents to distinguish between neoplastic and normal/healthy cells, since both follow the cell cycle phases. This lack of specificity is a serious limiting factor, resulting in serious adverse effects such as diarrhea, vomiting, nausea, alopecia, and myelosuppression [3].

In an attempt to minimize toxic effects and to improve the pharmacological performance of chemotherapeutic drugs, plant-based products (e.g., extracts, oils, and derived compounds) that were found to be effective and selective against tumor cells have been exploited either alone or in combination with conventional anticancers to improve chemotherapeutic treatment and to minimize the deleterious effects of treatment [4,5,6].

The association of drugs with herbs is a common practice to increase efficiency, reduce toxicity and even to improve taste. However, with the popularity of the use of plant-derived products as complementary treatments, there is a need to ensure the potential clinical benefits and identify these combinations [7,8].

Increasingly, the use of natural products in association with chemotherapeutics is becoming a current practice in cancer patients to stimulate the immune system [9,10].

Caetano et al. (2018) [11] characterized the use of medicinal plants in patients undergoing cancer treatments in the state of Sergipe, Brazil. Of the 331 patients interviewed, 49.55% reported the use of medicinal plants associated with conventional chemotherapeutic agents. Among these patients, 33 types of plants were identified and one of the most cited was *Matricaria recutita* L. (39.02%).

*Matricaria recutita* is a species that originated from Europe; however, it has been acclimatized in Asian and South American regions, being popularly known as German chamomile, Hungarian chamomile, or wild chamomile [12,13,14,15]. Teas and extracts are used for diarrhea, flatulence, spasms, inflammation, colitis, gastritis and hemorrhoids, anxiety, insomnia, and for reducing the risk of heart attack [16,17,18]. Although several of the claimed biological activities have already been demonstrated, the effects of the association of *Matricaria recutita* with antineoplastics are not yet fully disclosed.

In this study, we aimed to evaluate the antitumor effect and the toxicological parameters of the administration of the antineoplastic 5-fluorouracil associated with the aqueous *Matricaria recutita* flowers extract (MRFE) in an experimental model of sarcoma 180 tumor.

## 2. Materials and Methods

### 2.1. Vegetable Material

The commercial and organic samples of *Matricaria recutita* flowers were obtained from the company *Namastê Orgânicos* (Daterra Agroindustria Eireli, Sergipe, Brazil) in dehydrated form (Batch number C01211).

### 2.2. Production of Matricaria recutita Aqueous Extract

Flowers of *Matricaria recutita* were pulverized for 1 min using a blender to standardize the particle size [19]. Hot extraction by infusion was performed in a ratio of 3 g of crushed dry flowers to 150 mL of distilled water at approximately 100 °C according to the Brazilian Pharmacopoeia. The extract was lyophilized in a Liotop^®^ Model L101 Freeze Drying Machine (Liobras, São Carlos, São Paulo, Brazil) with a condenser temperature of −52 °C, 360 Vac, and 115 μHg. The total *Matricaria recutita* extract yield was calculated using the following equation:

$$EY=\frac{D_{ew}}{D_{lw}}\times 100$$

where *EY* is the total extract yield (%), *D_ew_* is the dry extract weight (g), and *D_lw_* is the dry flowers weight (g) [20].

### 2.3. Animals

A total of 42 Swiss male mice (weight 30 ± 5 g), obtained from the Bioterium of the Federal University of Sergipe, Brazil, were used. The animals were housed in cages with free access to food and water. All animals were kept under a 12/12 h light-dark cycle and were treated in accordance with the animal experimentation ethical principles of the Brazilian Association of Laboratory Animal Science (SBCAL). The in vivo experiments were approved by the Animal Studies Committee of the Federal University of Sergipe through the substantiated opinion on the experimental protocol (CEPA/UFS/60).

### 2.4. Cells

Cells from the experimental transplantable tumor called Sarcoma 180 (S180) or Crocker were obtained from the Oswaldo Cruz Foundation (Fiocruz, Bahia, Brazil) at the Tissue Engineering and Immunopharmacology Laboratory (LETI), and maintained in their ascitic form in the abdominal cavity of Swiss mice (Mus musculus), every 15 days, at the Pharmacology Laboratory of the inflammatory process at the Federal University of Sergipe (UFS) [21,22].

### 2.5. In Vitro Antitumor Activity Assay

For the in vitro antitumor activity assay, the S180 were used and plated into a 96-well plate (10^5^ cells/well suspended in 100 μL of medium) and evaluated by changes in the number of viable cells, as determined by the live cells capacity to reduce the yellow dye 3-(4,5-dimethylthiazol-2-yl)-2,5-diphenyl tetrazolium bromide (MTT) to a purple formazan product [23]. The MRFE (0.39–50 μg/mL) in vitro antitumor activity was individually evaluated. The 5-FU (0.06–4 μg/mL) activity was individually evaluated, or in association with the MRFE (50 μg/mL). The vehicle (DMSO) was used as a negative control. By the end of incubation (72 h), the plates were centrifuged, and the medium was then replaced by the medium (200 μL) containing 0.5 mg/mL of MTT. After three hours, the MTT-formazan product was dissolved in 150 μL of DMSO and the absorbance was measured at 595 nm using a multiplate reader (Biotek, ELx800 Absorbance Microplate Reader, Tecan, Männedorf, Switzerland).

### 2.6. In Vivo Antitumor Activity Assay

To assess the in vivo antitumor activity of the MRFE, 2 × 10^6^ sarcoma cells 180 per 0.5 mL were inoculated subcutaneously into the Swiss right axillary region. After 24 h, the animals were divided into six groups (*n* = 07 mice/group), undergoing treatments for seven consecutive days (once daily). The groups consisted of (i) Saline, as vehicle group (orally); (ii) 5-Fluorouracil 25 mg/kg/day (5-FU, purity > 99%; Sigma Chemical Co., St. Louis, MO, USA) as a positive control administered intraperitoneally (i.p.); (iii) MRFE 100 mg/kg/day (orally) as group test one; (iv) MRFE 200 mg/kg/day (orally) as group test two; (v) 5-FU 25 mg/kg/day associated with MRFE 100 mg/kg/day (i.p. and orally, respectively) as group test three; and (vi) 5-FU 25 mg/kg/day associated with MRFE 200 mg/kg/day (i.p. and orally, respectively) as group test four. At 24 h on the last day of treatment, under isoflurane anesthesia (inhalation: 1.5%, calibrated vaporizer), the animals were sacrificed by cervical dislocation and the tumors and organs were removed and weighed. The percentage of the tumor growth inhibition (TGI) was calculated by the following equation: TGI % = [(VG − TG)/CN] × 100, where VG is the mean tumor weight of the vehicle group, and TG is the median tumor weight of the test group [23].

### 2.7. Toxicological Analysis

The body mass of the animals was checked from the first to the last day of treatment. Changes in organ mass, hematology, and biochemistry (*n* = 07 mice/group) were determined at the end of the experiment. Prior to euthanasia, at 24 h after the last day of treatment, peripheral blood was collected from the retro-orbital plexus of the animals under inhalation anesthesia, (isoflurane 1.5%; calibrated vaporizer). After this procedure, the animals were euthanized and the livers, spleen, kidneys, heart, and tumors were removed, weighed, and dissected. The wet mass of each organ was expressed in grams per 100 g of body mass and compared to the vehicle group. For evaluation of the effect of 5-FU in association with MRFE, aspartate aminotransferase (AST), alanine aminotransferase (ALT), alkaline phosphatase (AP), urea, creatinine, and creatine kinase MB (CK-MB). Standard diagnostic kits from Biotechnology IND and (Varginha Ltd.a, Mato Grosso, Brazil) were used with a spectrophotometer. For hematological analyses, blood aliquots of the animals were placed in ethylenediaminetetraacetic acid (EDTA) for total and differential leukocyte counts, as determined by standard manual procedures using light microscopy.

### 2.8. Anatomic-Pathological and Histomorphological Analysis

Histological sections were made for microscopic analysis of the liver, kidney, spleen, and tumors. The hematoxylin and eosin (H/E) staining method was used. Upon animal euthanasia, viscera and tumors were stored in 10% formaldehyde, and then sliced and replaced in 10% formaldehyde for 24 h. Then, they underwent dehydration processing for 10 h in a PT09 TS Tissue Processor (LUPETEC™), followed by inclusion and paraffinization processing. Afterwards, the paraffin blocks were sliced using a 5 mm microtome and passed through the H/E staining process. After staining, the slides were assembled and fixed for further optical microscopy analysis. The histological areas of the tissues specified under study were recorded through photographs and stored for subsequent histopathological analysis. The mean number of mitoses/histological field (×400) was counted in the different experimental groups by the selection of 5 histological sections/tumor; and selection of 5 histological fields (400×)/section; being neglected areas of necrosis; the selected fields being randomized systematically: for each selected field, two ignored fields (left to right and top to bottom) followed. At any stage of the cell cycle (prophase, metaphase, anaphase, and telophase), typical and atypical mitotic figures were counted.

### 2.9. Tumor Cell Proliferative Index Analysis Using In Situ Ki67 Antigen Immunodetection

Histological sections (3 μm thickness), previously salinized and subjected to the immunohistochemical reaction, were assembled on glass slides using the indirect streptavidin-biotin method. The slides were dewaxed in xylol and washed with lessening concentrations of ethyl alcohol (100%, 95%, 90%, 80%, and 70%). Antigenic recovery was performed by immersing the sections in citrate solution, being heated for 20 min under microwave to potentiate the reaction. Marking of the Ki-67 antigen was performed by incubation with rabbit anti-mouse MIB-1 monoclonal antibody (Dako, Glostrup, Denmark, at 1:50 dilution) for 30 min. The revealing reaction was performed with the use of diaminobenzidine (DAB, Ventana Medical Systems, Tucson, AZ, USA), and counter-colored with Meyer’s Hematoxylin. The two steps were completed in four minutes each. The positive control was performed using human tonsil. For the negative control, the primary antibody in the reaction was substituted with phosphate buffered saline. The positivity of the immunostaining was confirmed from the brownish hued cellular precipitate. Thus, cells with immunolabelled nuclei, being stained in brown, regardless of the labeling intensity, were evaluated as positive. The immunoexpression gradation was established from the percentage of positive cells per 1000 cells counted.

### 2.10. Statistical Analysis

Distinctions between the experimental groups were compared using ANOVA (analysis of variance), followed by the Student–Newman–Keuls test (*p* < 0.05); and ANOVA (analysis of variance), followed by the Tukey post test (*p* < 0.05). Statistical analyses were performed using the GraphPad program (Intuitive Software for Science, San Diego, CA, USA).

## 3. Results and Discussion

*Matricaria recutita* is widely used in teas and infusions for the treatment of many health disorders [24], including patients undergoing chemotherapy treatment [11]. Thus, in this study, antitumor activity of 5-FU combined with MRFE was assessed to evaluate the synergistic effect of this association.

In the evaluation of the antitumor activity in vitro against the Sarcoma 180 cell line, MRFE did not present cytotoxicity in the tested concentrations (Table 1). The cytotoxic activity of MRFE in a range of tumor cell lines has however been documented, e.g., in human liver cancer cells (HEpG2) [13], human breast cancer cells (MDA-MB-468 and MCF-7) [25], and human prostate cancer cells [26]. In all these studies, however, the IC_50_ was greater than 50 µg/mL and, to be promising candidates for antineoplastic activity in vivo, the screening program recommend that extracts and oils must have IC_50_ lower than 30 µg/mL [5]. In a study by Bijak et al. (2013), *Matricaria recutita* was tested up to 50 µg/mL against mouse fibroblast cultures L929 and human lung cells A549. The fact there were no signs of cytotoxicity [27] corroborate our findings.

When evaluating the results of 5-FU alone and in association (5-FU+MRFE), high cytotoxicity was recorded, but without statistically significant differences (Table 1). This is indicative that the MRFE does not influence the antitumor activity in vitro against sarcoma 180 at the tested concentrations. No studies were yet found in the literature the in vitro beneficial effects of the association of MRFE with 5-FU. Ogata-Ikeda et al. (2011) [28] reported, however, that bisaboloxide A (one of the constituents isolated from *Matricaria recutita*) potentiated the growth inhibition of the leukemic cell line (K562) when associated with 5-FU. The authors still suggest that the simultaneous application of *Matricaria recutita* may reduce the dose of 5-FU required in chemotherapeutic treatments [28]. Our results, however, demonstrate that this association does not seem to be favorable for the potentiation of antineoplastic activity in vitro (Table 1).

**Figure 1:**
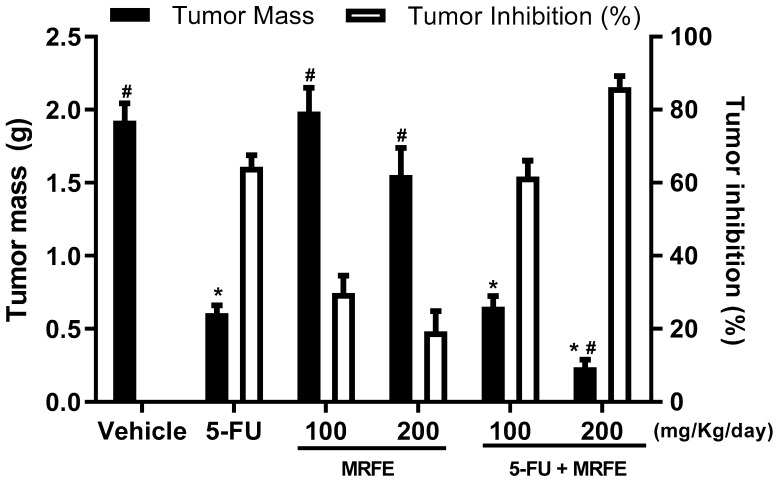
In vivo antitumor effect of 5-FU 25 mg/kg/day associated with MRFE 200 mg/kg/day in animals transplanted with sarcoma 180. The vehicle group was treated with saline and the 5-FU group with 5-Fluorouracil 25 mg/kg/day. The values correspond to the mean ± S.E.M. of 07 animals/group analyzed using one-way analysis of variance with the Student–Newman–Keuls post test. * *p*< 0.05 compared to the vehicle group. ^#^ *p* < 0.05 compared to the 5-FU group.

As no report was found in the literature on the evaluation of the anti-tumor activity of MRFE in an in vivo sarcoma 180 model, we tested the anti-tumor activity of the isolated MRFE at two distinct doses (100 and 200 mg/kg/day). No significant anti-tumor activity was reported. However, 5-FU at a dose of 25 mg/kg/day alone, and the association of 5-FU 25 mg/kg/day with the MRFE 100 or 200 mg/kg/day, significantly reduced the average tumor mass (*p* < 0.05) with tumor inhibition of 64.3%, 66.7%, and 87.7%, respectively, when compared to vehicle group. When assessing the association of 5-FU with MRFE in both tested doses with isolated 5-FU, only a reduction in tumor mass was observed for the combination of 5-FU 25 mg/kg/day with MRFE 200 mg/kg/day, with a tumor inhibition rate of 86.1% (Figure 1).

These data suggest that the association of 5-FU with MRFE at a dose of 200 mg/kg/day may improve the effect of 5-FU alone, contributing synergistically for the tumor reduction. In the clinic, the combination of antineoplastics (polychemotherapy) is a common practice to minimize problems related to the use of a single medication (less efficacy, increased dose, greater toxicity, and tumor resistance) [29,30].

Research shows that natural products are becoming a common practice in cancer therapy due to their synergistic actions with antineoplastic drugs, as demonstrated by Amaral et al. (2016) [21] and Bezerra et al. (2006) [23], who reported significant results from the association of *Mentha x villosa*, piplartine, and piperine with the 5-FU antineoplastic in a Sarcoma 180 model [21,23].

Macroscopically, the excised tumors formed irregular and voluminous solid masses in the vehicle, MRFE 100, and MRFE 200 groups, but showed a significant reduction in their mass in the 5-FU alone, 5-FU associated with MRFE 100, and even greater reduction in 5-FU associated with MRFE 200 groups.

**Figure 2:**
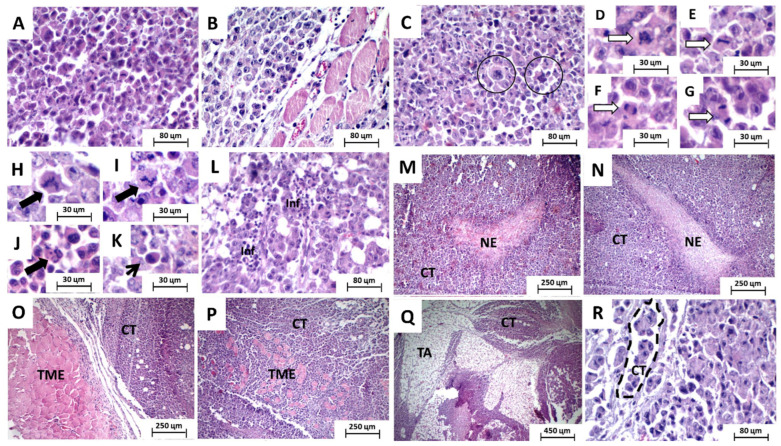
Photomicrographs of HE-stained histological sections of surgical specimens of sarcoma 180 implanted in rodents. Circles—giant tumor cells with bizarre nuclei; white arrows—typical mitotic figures; dark arrows—atypical figures; thin arrow—apoptotic bodies; dashed line—presence of tumor cell emboli within blood vessels A-C, Rx400, D-K x800, and M-Q x100. Caption: CT—viable tumor cells; NE—coagulative necrosis; TME—skeletal striated muscle tissue; TA—subcutaneous adipose tissue. (**A**,**B**) prominent nucleoli; (**C**) tumor giant cells with hyperchromatic misshapen nuclei; (**D**) prophase; (**E**) metaphase; (**F**) anaphase; (**G**) telophase; (**H**) atypical tripolar spindles; (**I**) atypical tetrapolar spindles; (**J**) atypical prophases; (**K**) apoptotic bodies; (**M**,**N**) comedo-type necrosis; (**L**) peri and intratumoral inflammatory infiltrate rich in lymphocytes; (**O**) tumor cells compressing the peripheral skeletal muscle tissue; (**P**) tumor cells invading and dissociating skeletal muscle tissue; (**Q**) tumor cells permeating and disorganizing the subcutaneous adipose tissue; (**R**) tumor cell emboli within blood vessels.

Under microscopic analysis, sarcoma 180 specimens were represented by proliferation of polyhedral, cuboid, and occasionally ovoid cells arranged in compact sheets, with little intervening stroma, supported by a delicate vasculocapillary network. Individually, the neoplastic cells showed broad eosinophilic cytoplasm with well-defined limits and voluminous nuclei, sometimes hyperchromatic, sometimes showing finely dispersed chromatin, and one to two prominent nucleoli (Figure 2A,B). Some tumor giant cells with bizarre and hyperchromatic misshapen nuclei could eventually be observed (Figure 2C). Mitotic figures were abundant and exhibited predominantly typical morphology in all phases (prophase—Figure 2D, metaphase—Figure 2E, anaphase—Figure 2F and telophase—Figure 2G), although atypical mitotic figures (such as, tripolar—Figure 2H and tetrapolar spindles—Figure 2I and atypical prophases—Figure 2J were common) could occasionally be observed, as well as some apoptotic bodies (Figure 2K).

Amidst the parenchymal tumor component, extensive areas of coagulative necrosis (comedo-type necrosis—Figure 2M,N) could be seen, structured in the form of trabeculae (sometimes giving the proliferative component a pseudolobular arrangement) or forming irregular blocks within the tumor sheets. The tumor margins were irregular and poorly defined in some cases, giving a more infiltrative aspect to the tumor, but were quite regular and well defined in others, giving an appearance of tumor growth by expansion. In general, the stroma was quite sparse and composed of delicate fibrous connective tissue. Discrete peri and intratumoral inflammatory infiltrate, rich in lymphocytes, was also observed (Figure 2L). Tumor cells were also observed compressing the peripheral skeletal muscle tissue (Figure 2O) and sometimes invading and dissociating this tissue (Figure 2P). They were also observed permeating and disorganizing the subcutaneous adipose tissue (Figure 2Q). The presence of tumor cell emboli within blood vessels was also observed (Figure 2R—dashed line).

**Figure 3:**
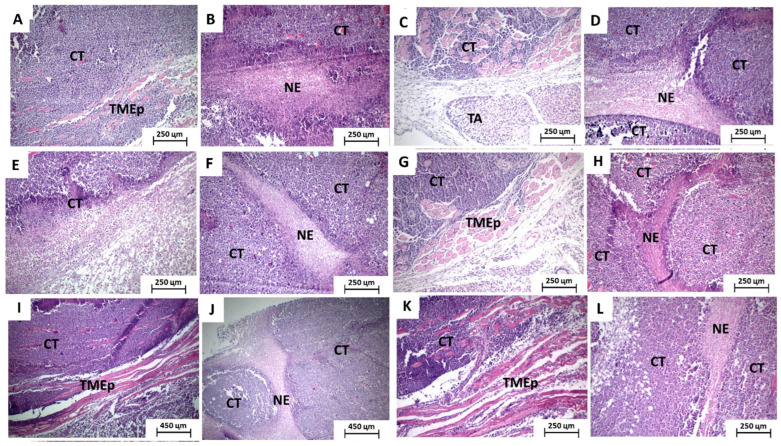
Photomicrographs of HE-stained histological sections of surgical specimens of sarcoma 180 implanted in rodents. (**A**) vehicle group showing tumor with infiltrative and irregular margins, invading and dissociating peripheral muscle tissue and (**B**) forming solid intraparenchymal blocks of coagulative necrosis. (**C**) 5-FU group showing regular tumor margins, with a more expansive growth pattern and (**D**) thin bands of coagulative necrosis forming a trabecular pattern. (**E**) Group treated with 100 mg/kg of the extract showing very infiltrative tumor margins and (**F**) forming thick intraparenchymal bands of coagulative necrosis. (**G**) Group treated with 200 mg/kg showing much more regular tumor margins and (**H**) trabeculae of intratumoral coagulative necrosis. (**I**,**J**) Group treated with 5-FU associated with 100 mg/Kg and (**K**,**L**) 200 mg/kg of the extract exhibiting regular tumor margins compressing peripheral skeletal striated muscle tissue and thin trabeculae of intratumoral coagulative necrosis. Caption: CT—viable tumor cells; NE—coagulative necrosis; TMEp—skeletal striated muscle tissue; TA—subcutaneous adipose tissue (×100).

When analyzed separately, some differences between the groups were observed. The saline-treated group exhibited intense cytological atypia, massive invasion, dissociation, and destruction of skeletal striated muscle fiber bundles and formation of extensive areas of necrosis forming intraparenchymal solid blocks. The tumors showed irregular infiltrative margins, permeating and invading the surrounding fibrous and fatty subcutaneous tissue (Figure 3A,B). The group treated with 5-FU had more regular tumor margins, with less peripheral infiltration, and the areas of coagulative necrosis were thin and formed intraparenchymal trabeculae that gave the tumor a pseudolobular appearance (Figure 3C,D).

The group treated with 100 and 200 mg/Kg of the extract exhibited morphological characteristics very similar to those observed in the saline group, expressed by infiltrative and irregular tumor margins and coagulative necrosis forming solid intratumoral sheets (Figure 3E–H). Furthermore, both groups treated with the association between 5-FU and extract, at the two doses tested (100 and 200 mg/kg), presented histopathological characteristics very close to those observed in the 5-FU group alone (Figure 3I–L).

**Figure 4:**
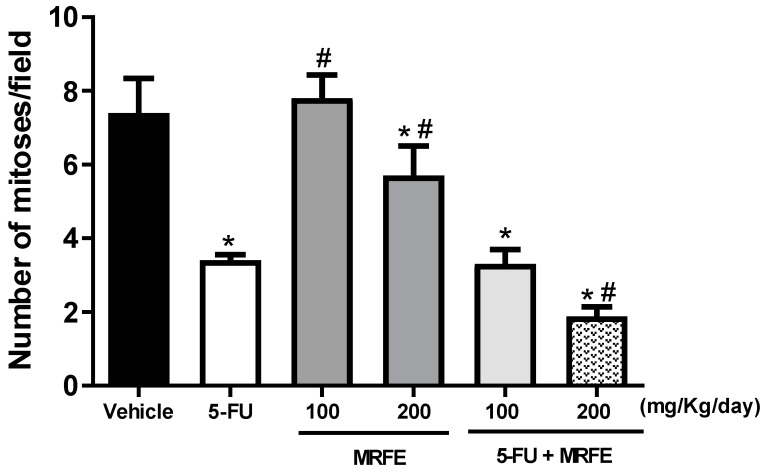
Quantitative analysis of the mean mitosis/histological field (×400) for the different experimental groups. Data expressed as mean ± S.E.M. Data analyzed by one-way analysis of variance (ANOVA) with Tukey post test. * *p* < 0.05 compared to the vehicle group. ^#^ *p* < 0.05 compared to the 5-FU group.

In the semiquantitative analysis of histopathological parameters of tumor aggressiveness, Figure 4 demonstrates the mean number of mitoses/histological field analyzed among the treated groups. The MRFE 100 groups did not differ significantly from the vehicle group. The MRFE 200, 5-FU group alone, and the groups associated with MRFE 100 and 200 mg/kg differed significantly from the vehicle group. However, 5-FU associated with MRFE 200 showed a statistical difference when compared to 5-FU alone; data corroborated the assumption of a possible positive interference of MRFE 200 in the antineoplastic activity of 5-FU, as depicted in Figure 1.

**Figure 5:**
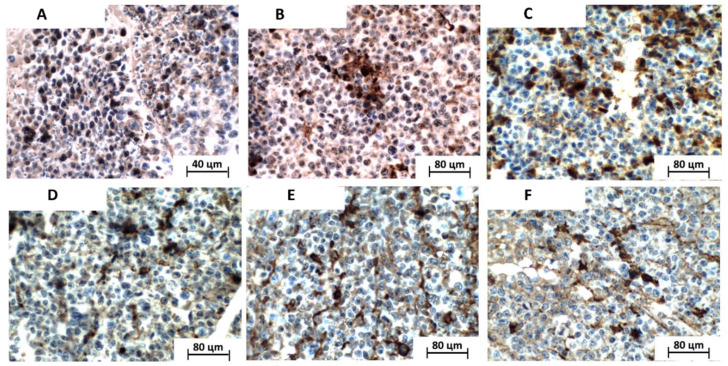
Photomicrographs of histological sections demonstrating immunohistochemical expression of the Ki67 antigen (brownish staining) in the tumors analyzed. The tumor cells presented predominantly nuclear positivity (LSAB, 400×). (**A**) vehicle group; (**B**) 5-FU group; (**C**) MRFE 100 group; (**D**) MRFE 200 group; (**E**) 5-FU+MRFE 100 group; (**F**) 5-FU+MRFE 200 group.

Regarding quantitative analysis of the Ki67 proliferation antigen, all the analyzed cases presented diffuse Ki67 antigen immunoreactivity, evidenced mainly in the cell nuclei, although in varying proportions. A greater marking was observed in the solid sheet central portions of the tumor cells, as well as at the invasive front, over the detriment of regions adjacent to necrotic areas (Figure 5).

**Figure 6:**
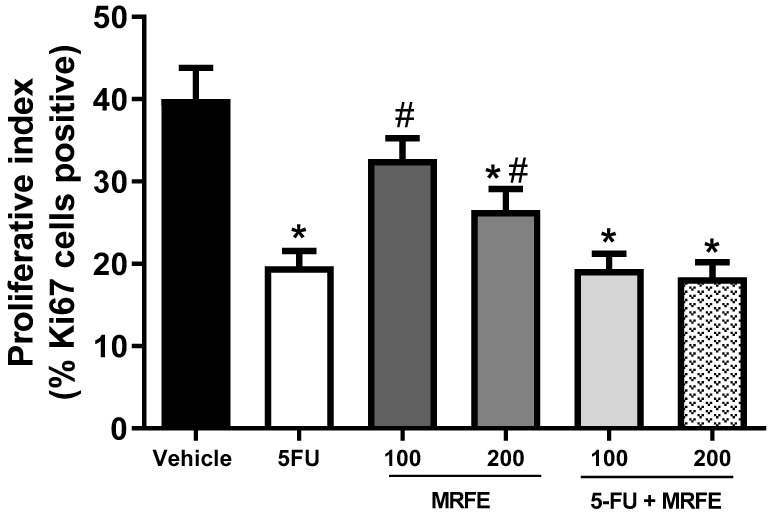
Quantitative analysis of Ki67 positive cells (nuclear immunostaining) representative of the estimation of the tumor proliferative index in the different experimental groups. Significant differences from the saline group were expressed as * *p* < 0.05, significant differences from the 5-FU group were expressed as ^#^ *p* < 0.05 (ANOVA and Bonferroni multiple comparison post test).

In the quantitative analysis of the proliferative index, the number of Ki67-positive cells, interpreted as cycling cells, was significantly lower in the groups treated with isolated 5-FU (19.6 ± 1.9 %), MRFE 200 (26.5 ± 26.0%), 5-FU associated with MRFE 100 (9.33 ± 1.9%), and 5-FU associated MRFE 200 (18.33 ± 1.8%) than in the vehicle-only group (40.0 ± 3.8). However, there was no difference in the cellular proliferative index between the vehicle group and the MRFE 100 group (32.7 ± 2.5%) (Figure 6). Quantitative analysis of in situ Ki67 antigen immunoexpression has been used to evaluate the biological behavior of tumors in preclinical experiments associated with the antitumor activity of natural substances in research with sarcoma 180 [31,32].

The joint analysis of these data (Figure 1, Figure 4 and Figure 6) is indicative that the association of 5-FU with MRFE 200 may enhance the antitumor activity of the antineoplastic agent. This result can be explained by the fact that treatment with MRFE 200 mg/kg/day alone (Figure 5 and Figure 6) reduces the number of mitoses in the histopathological analysis of sarcoma 180.

5-FU is the third most commonly used chemotherapeutic agent worldwide in the treatment of solid tumors; however, its application is severely limited because of its hepatic, renal, cardiac, and hematopoietic toxicity [33]. Thus, based on the increased antitumor effect due to the association of 5-FU 25 with MRFE 200 mg/kg/day, possible changes in the systemic toxicological aspects of this combination were investigated in mice with Sarcoma 180.

The first set of evaluated toxicological aspects were the variation of the body and visceral mass (brain, heart, intestine, kidneys, lung, liver, spleen, and stomach). Significant reduction was observed in body mass in the groups treated with 5-FU isolated and 5-FU combined with MRFE 200 mg/kg/day, when compared with the vehicle group. However, greater loss of body mass, in addition to diarrhea with soft stools, occurred in the group treated with 5-FU associated, when compared with 5-FU isolated (Table 2).

Our data confirm that associating 5-FU with MRFE 200 mg/kg/day promotes the progressive loss of body mass throughout the treatment. The animals treated with the association had intense diarrhea, a fact that may have caused the loss of body mass in the animals. Clinically, such a result could have dramatic consequences for the patient [34]. Both therapeutic and toxic effects depend on the time of exposure and the concentration of the drug present in the plasma; in addition, toxicity depends on the type of body tissue [35,36,37]. Thus, the potentiation of chemotherapeutic drugs may aggravate the clinical outcome of patient.

According to Yu et al. (2018) [38], weight loss is usually associated with toxicity and can be analyzed by varying organ mass or reduced consumption of food and water. Our results show that the treatment with 5-FU isolated and 5-FU combined with MRFE 200 produced significant changes only in the spleen. The animals treated with the isolated 5-FU and 5-FU+MRFE 200 associated group significantly reduced spleen mass when compared to the vehicle group, however without changes when taken alone (Table 2). This indicates that the MRFE 200 associated with 5-FU did not worsen the spleen toxicity observed at the macroscopic level. Spleen atrophy is a side effect resulting from the use of most chemotherapy drugs, including 5-FU, which ends up in the immunosuppressive activity of the drug, which is a limiting feature for therapy, as it increases susceptibility to infections [39].

Because 5-FU has the potential to affect hepatic, renal, and cardiac function, biochemical tests were performed in order to functionally evaluate these organs. Table 3 shows the results obtained for 5-FU and MRFE activity, alone and combined, in the assessment of liver (ALT, AST, and AP), renal (urea and creatinine), and cardiac function (CK-MB). According to the results obtained from the evaluation of biochemical parameters, no changes in renal and cardiac function were observed. Although 5-FU can induce kidney problems, the time of exposure to the antineoplastic seems not to have been sufficient to cause nephrotoxicity [40].

When analyzing liver function, no significant changes were found (Table 3). On the other hand, when analyzing the AST, a significant reduction of the enzyme was found in the 5-FU group alone and associated with MRFE 200. This result is justified by the fact that AST is also found in skeletal and cardiac muscles, kidneys, pancreas, and erythrocytes. The S180 tumor, in its solid form and as it grows in the axillary region, damages one or more tissues, such as skeletal muscle. This factor causes changes in biochemical parameters, such as AST [21]. For this reason, care should be taken with the isolated elevation of AST, as it does not correspond in most cases to liver damage. Therefore, the decrease in AST, shown in the groups treated with 5-FU and associated with MRFE 200 when compared to the vehicle group, may not be related to liver damage, but it is possibly linked to the decrease in tumor growth. In specific models of hepatotoxicity induction, *Matricaria recutita* has demonstrated hepatoprotective activity though [41,42].

Chemotherapeutic agents, including 5-FU, commonly cause hematological changes; therefore, the effect of the association of 5-FU and MRFE 200 on hematological parameters was also evaluated. The reported results demonstrate that the association between 5-FU and MRFE 200 did not induce changes in the parameters hemoglobin, erythrocytes, and hematocrit. However, when platelets and leukocytes were evaluated, changes were observed (Table 4).

Despite the significant change in the number of platelets, the occurrence of thrombocytopenia in the 5-FU groups alone and in association, in relation to the vehicle group, demonstrates that the association did not promote the setup of adverse side effects. Chemotherapeutic agents are quite aggressive against bone marrow, the cells of which are very sensitive, and myelosuppression is one of the side effects of 5-FU [43]. Thus, thrombocytopenia may have resulted from myelosuppression caused by the action of the isolated and associated drug, but without potentiating the effect.

Furthermore, Table 4 shows that the groups 5-FU isolated and 5-FU associated with MRFE 200 resulted in significant changes in the total number of leukocytes when compared to the vehicle group. This result highlights the reduction in total leukocytes for isolated and associated treatment, in relation to the vehicle group. In addition, it is noteworthy that the 5-FU isolated group was as subject to infections as the associated 5-FU group. From this, it is evident that the association did not potentiate the short-term leukopenia induced by antineoplastic. In addition, the percentage ratio of neutrophils and lymphocytes are also found to be altered; however, the association of 5-FU with MRFE 200 does not seem to interfere with this alteration. Such results do not corroborate the work by Amaral et al. (2016) [21] and Bezerra et al. (2006) [23], as the authors obtained positive results with the associations between 5-FU and *Mentha x villosa* oil, and 5-FU and piplartine, respectively, decreasing the adverse effects of leukopenia, when compared to 5-FU isolated (10 mg/kg/day) [21]. In both associations, there was an improvement in leukopenia, including reversal.

As reported for the toxicological parameters, anatomopathological and histomorphological analyzes also showed alterations only in the spleen. Splenic tissue exhibited extensive lymphoid follicles with variable diameters characterized by white pulp surrounded by red pulp in the vehicle- and MRFE 200-treated groups. This same area was reduced in the isolated 5-FU and 5-FU+MRFE treated groups, with spleen tissues presenting fewer lymphoid follicles. The reduction in the white pulp in these groups, with consequent splenic atrophy, corroborates the findings by Amaral et al. (2016) [21], in which such suppression in the spleen with antineoplastic treatment was also observed.

**Figure 7:**
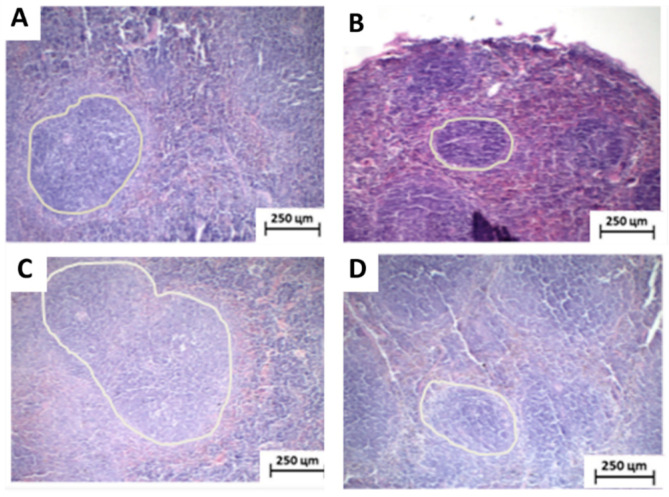
Photomicrographs of HE-stained histological sections of surgical specimens of spleen from rodents with sarcoma 180. (**A**) Vehicle group and (**B**) MRFE group 200 mg/kg/day, demonstrating extensive white hair region. (**C**) Group 5-FU alone and (**D**) Group 5-FU associated with MRFE 200 mg/kg/day, showing atrophy of the white fur. (×100).

In the analysis of data on spleen mass (Table 2), number of leukocytes (Table 4), and histological sections (Figure 7), it is possible to conclude that the MRFE 200 mg/kg/day does not interfere with the immunosuppression caused as an adverse effect of 5-FU.

## 4. Conclusions

Based on the obtained results, it is strongly suggested that the antineoplastic 5-FU associated with MRFE 200 mg/kg/day markedly potentiates the antitumor action of the drug. However, no benefit was observed from the association related to hematological, biochemical, histopathological parameters, and variations in body and organ mass. Together, these data suggest that the associated use may be safe for patients being treated with 5-FU; however, the study draws attention to the observation of diarrhea and consequent greater loss of body mass. Thus, long-term studies reporting the association of classical chemotherapeutics with naturally occurring anticancers are promising.

## Figures and Tables

**Table 1:** Evaluation of MRFE in the interference in the in vitro antitumor activity of the 5-FU.

Cell Line	IC_50_ µg/mL		
	MRFE	5-FU	5-FU+MRFE
Sarcoma 180	>50	1.5 ± 0.8	1.1 ± 0.6

MRFE: *Matricaria recutita* flowers extract; 5-FU: 5-fluorouracil. Data are displayed as maximum inhibitory concentration capable of providing 50% of maximum effect (IC_50_) ± standard error of the mean (SEM) values performed in triplicate in three different experiments different measured by the MTT assay after 72 h of incubation.

**Table 2:** Effect of 5-FU associated with MRFE 200 mg/kg/day on body and visceral mass variation in animals transplanted with sarcoma 180.

	Vehicle	5-FU (25 mg/kg/day)	MRFE (200 mg/kg/day)	5-FU+MRFE (25 + 200 mg/kg/day)
Variation in body mass (g)	3.451 ± 0.5295	−5.911 ± 0.7351 *	4.799 ± 1.026	−7.09 ± 0.5283 *^#^
Brain mass (g/100 g)	1.11 ± 0.05	1.31 ± 0.07	1.12 ± 0.02	1.27 ± 0.06
Heart mass (g/100 g)	0.45 ± 0.03	0.40 ± 0.01	0.42 ± 0.02	0.48 ± 0.02
Intestine mass (g/100 g)	10.13 ± 0.49	11.71 ± 0.57	11.02 ± 0.41	9.99 ± 0.69
Kidneys mass (g/100 g)	1.27 ± 0.03	1.14 ± 0.01	1.43 ± 0.09	1.18 ± 0.07
Lung mass (g/100 g)	0.60 ± 0.03	0.60 ± 0.04	0.65 ± 0.05	0.59 ± 0.01
Liver mass (g/100 g)	4.92 ± 0.11	4.20 ± 0.19	4.78 ± 0.22	4.15 ± 0.19
Spleen mass (g/100 g)	0.69 ± 0.02	0.23 ± 0.02 *	0.68 ± 0.02	0.21 ± 0.02 *
Stomach mass (g/100 g)	1.06 ± 0.05	0.86 ± 0.03	0.91 ± 0.05	0.99 ± 0.06

The data correspond to the mean ± S.E.M. of 07 animals/group studied using one-way analysis of variance (ANOVA) with the Student–Newman–Keuls post test. * *p* < 0.05 compared to the vehicle group. ^#^ *p* < 0.05 compared to the 5-FU/MRFE (100 mg/kg/day) associated group.

**Table 3:** Effect of 5-FU associated with MRFE on biochemical parameters in animals transplanted with S180 tumor.

	Vehicle	5-FU (25 mg/kg/day)	MRFE (200 mg/kg/day)	5-FU+MRFE (25 + 200 mg/kg/day)
ALT (U/L)	65.31 ± 5.9	61.5 ± 4.6	48.5 ± 4.6	67.0 ± 7.3
AST (U/L)	260.7 ± 25.1	168.8 ± 28.9 *	224.4 ± 19.4	155.4 ± 17.6 *
AP (U/L)	40.6 ± 3.2	30.2 ± 9.3	42.6 ± 5.3	35.8 ± 8.7
Urea (mg/dL)	36.7 ± 2.2	35.0 ± 4.6	33.4 ± 3.2	36.4 ± 4.0
Creatinine (mg/dL)	2.0 ± 0.1	2.1 ± 0.1	1.9 ± 0.07	2.2 ± 0.1
CK-MB(U/L)	8.8 ± 1.4	9.3 ± 2.9	8.1 ± 2.7	9.9 ± 3.7

The data correspond to the mean ± SEM of animals studied using one-way variance analysis (ANOVA) with the Student–Newman–Keuls post test. * *p* < 0.05 compared to the vehicle group.

**Table 4:** Effect of 5-FU associated with MRFE on hematological parameters in animals transplanted with S180.

	Vehicle	5-FU (25 mg/kg/day)	MRFE (200 mg/kg/day)	5-FU+MRFE (25 + 200 mg/kg/day)
Hemoglobin g/dL	11.6 ± 0.1	12.3 ± 0.3	12.0 ± 0.2	12.2 ± 0.5
Erythrocite 10^6^/UL	6.5 ± 0.2	6.7 ± 0.3	5.7 ± 0.3	6.5 ± 0.1
Hematocrit %	31.3 ± 0.9	32.4 ± 1.5	29.9 ± 1.9	32.4 ± 0.8
Plaquetas 10^3^ /dL	498.1 ± 16.5	221.0 ± 23.6 *	446.7 ± 30.1	266.4 ± 30.4 *
Total Leukocytes (10^3^ cels/µL)	5.7 ± 0.2	0.9 ± 0.07 *	5.8 ± 0.3 ^#^	0.7 ± 0.06 *
Neutrophils %	36.1 ± 3.5	24.6 ± 2.0 *	39.4 ± 2.1 ^#^	22.7 ± 1.7 *
Eosinophils %	1.1 ± 0.3	0.6 ± 0.2	0.6 ± 0.2	0.3 ± 0.2
Lymphocytes %	56.0 ± 3.8	81.2 ± 2.7 *	50.9 ± 2.9 ^#^	75.5 ± 2.8 *
Monocytes %	1.9 ± 0.4	1.9 ± 0.7	1.3 ± 0.4	2.5 ± 0.5

The data correspond to the mean ± SEM of animals studied using one-way variance analysis (ANOVA) with the Student–Newman–Keuls post test. * *p* < 0.05 compared to the vehicle group. ^#^ *p* < 0.05 compared to the 5-FU group (25 mg/kg/day).

## Data Availability

Data are available from authors upon request.
